# Overexpression of RACK1 Promotes Metastasis by Enhancing Epithelial-Mesenchymal Transition and Predicts Poor Prognosis in Human Glioma

**DOI:** 10.3390/ijerph13101021

**Published:** 2016-10-18

**Authors:** Qiao-Li Lv, Yuan-Tao Huang, Gui-Hua Wang, Yan-Ling Liu, Jin Huang, Qiang Qu, Bao Sun, Lei Hu, Lin Cheng, Shu-Hui Chen, Hong-Hao Zhou

**Affiliations:** 1 Department of Clinical Pharmacology, Xiangya Hospital, Central South University, Changsha 410008, China; lvqiaoli2008@sina.com (Q.-L.L.); 13487569804@163.com (Y.-L.L.); guoying198688@163.com (J.H.); scy_csu2016@163.com (B.S.); hu773589905@163.com (L.H.); 2 Hunan Key Laboratory of Pharmacogenetics, Institute of Clinical Pharmacology, Central South University, Changsha 410078, China; 3 Department of Neurology, The Brain Hospital of Hunan Province, Changsha 410008, China; m13397602527@163.com; 4 Department of Oncology, Changsha Central Hospital, Changsha 410008, China; zhuifenglang12@126.com; 5 Department of Pharmacy, Xiangya Hospital, Central South University, Changsha 410008, China; quqiang1983@sina.com; 6 State Key Laboratory of Ophthalmology, Zhongshan Ophthalmic Center, Sun Yat-sen University, Guangzhou 510275, China; kjade.cheng@hotmail.com

**Keywords:** RACK1, cell cycle, epithelial-mesenchymal transition, prognosis

## Abstract

Emerging studies show that dysregulation of the receptor of activated protein kinase C1 (RACK1) plays a crucial role in tumorigenesis and progression of various cancers. However, the biological function and underlying mechanism of RACK1 in glioma remains poorly defined. Here, we found that RACK1 was significantly up-regulated in glioma tissues compared with normal brain tissues, being closely related to clinical stage of glioma both in mRNA and protein levels. Moreover, Kaplan-Meier analysis demonstrated that patients with high RACK1 expression had a poor prognosis (*p* = 0.0062, HR = 1.898, 95% CI: 1.225–3.203). In vitro functional assays indicated that silencing of RACK1 could dramatically promote apoptosis and inhibit cell proliferation, migration, and invasion of glioma cells. More importantly, knockdown of RACK1 led to a vast accumulation of cells in G0/G1 phase and their reduced proportions at the S phase by suppressing the expression of G1/S transition key regulators Cyclin D1 and CDK6. Additionally, this forced down-regulation of RACK1 significantly suppressed migration and invasion via inhibiting the epithelial-mesenchymal transition (EMT) markers, such as MMP2, MMP9, ZEB1, N-Cadherin, and Integrin-β1. Collectively, our study revealed that RACK1 might act as a valuable prognostic biomarker and potential therapeutic target for glioma.

## 1. Introduction

Glioma, accounting for about 70% of malignant tumors in the central nervous system (CNS), is the most primary and lethal brain tumor [1,2,3]. In particular, patients with Glioblastoma, the most aggressive form of malignant glioma, only have a median survival time of approximately 14 months [4]. Despite advances in treatment strategy, such as surgery combined with chemo-radiotherapy, there is generally no significantly improved clinical outcomes for glioma patients mainly due to their resistance to chemo-radiotherapy and insufficient understanding of the molecular mechanisms of glioma [5,6]. Moreover, similar histological features of glioma may exhibit different clinical characteristics and response to therapy [7]. Therefore, it is crucial to unravel the underlying molecular mechanisms and explore potential prognostic biomarkers, which contributes to the development of new effective therapeutic targets and strategies for glioma.

The receptor of activated protein kinase C1 (RACK1, GNB2L1) was initially discovered as an anchoring protein for activating protein kinase C (PKC) [8]. As a scaffold protein, RACK1 was composed of seven internal Trp-Asp 40 (WD40) repeats, which was similar to a bladed propeller structure [9]. Intriguingly, this structure allowed RACK1 to interact with a variety of proteins and exert diverse functions. Increasing evidence indicates that RACK1 acts as a versatile signaling pathway hub, providing a platform for protein-protein interaction [10], which plays a crucial role in a diverse number of cellular biological processes, including signal transduction, virus infection, immune response, neural development, and cell differentiation [11,12,13,14]. Emerging studies show that dysregulated RACK1 is supposed to be a biomarker or regulator in a wide spectrum of human cancers [15,16,17,18,19,20,21], which implies that RACK1 could play an important role in genesis and progression [18,22] as well as resistance to chemotherapy in tumors [23]. In addition, Wang et al. and Choi et al. reported that overexpression of RACK1 strongly was linked with advanced clinical stage and poor prognosis in esophageal squamous cell carcinoma and non-small cell lung cancer [24,25]. However, the implication of RACK1 in glioma progression was still unclear. In the present study, we investigated the relationship of RACK1 with clinical characteristics as well as prognosis of glioma patients. Moreover, we examined the effect and underlying molecular mechanisms of RACK1 in the regulation of the cell cycle, apoptosis, migration, and invasion in glioma U87 and U251 cells.

## 2. Materials and Methods

### 2.1. Patients and Follow-Up

This study was approved by the ethics committee of The First Affiliated Hospital of Nanchang University (Ethical Approval No. 2010-015; Date: 12 March 2010) with informed consent obtained from each patient before this study. 

A total of 173 glioma tissues were obtained from The First Affiliated Hospital of Nanchang University (Nanchang, China) between November 2010 and June 2013, with no patients receiving anticancer therapy or radiotherapy before tumor surgical resection. Of the patients, 92 had experienced follow-up time lasting 48 months since the date of surgical resection. Overall survival (OS) was defined as the period of time between the date of the initial surgical operation and death or the last follow-up. Twenty normal brain tissues were from patients with surgical procedures for epilepsy or craniocerebral injuries, which required partial resections of brain tissue to reduce intracranial pressure.

### 2.2. Cell Culture and siRNA Interference

U87 and U251 human glioma cell lines were purchased from the Cell Bank of the Shanghai Branch of Chinese Academy of Sciences, and cultured in Dulbecco’s modified Eagle’s medium (DMEM) with 10% fetal bovine serum (FBS). All cells were maintained at humidified 37 °C incubator with 5% CO_2_. Two RACK1-specific siRNAs, one targeting position 5’-CAAACACCTTTACACGCTA-3’, named siRNA1-RACK1 and the other targeting position 5’-CAGGGATGAGACCAACTAT-3’, named siRNA2-RACK1, as well as negative control (non-specific scramble siRNA) were synthesized by Ribobo (Guangzhou, China).

Cells (60% confluent) were transfected with 100 nM siRNA using 6 μL Lipo-RNAiMAX following the manufacturer’s instruction (Invitrogen, Carlsbad, CA, USA), and then the effectiveness of RACK1-siRNA was detected by both quantitative real-time polymerase chain reaction (qRT-PCR) and Western blot analysis.

### 2.3. Cell Proliferation Assays

The effect of siRNA-RACK1 on the proliferation of glioma cells was evaluated using CellTiter 96 aqueous one solution reagent (MTS) assays. U87 and U251 cells were seeded in a 96-well plate with 3 × 103 cells per well, the cells were divided into four groups, and each group had six wells. After cells attached, they were transfected with NC or siRNA-RACK1, and cultured for 24, 48, 72, and 96 h. The medium was then replaced by 100 µL MTS and DMEM medium with a ratio of 1:9. Cell viability was then obtained by measuring the absorbance at a wavelength of 490 nm. All experiments were performed at least three times.

### 2.4. Colony Formation Assay

U87 and U251 cells were transfected with NC or siRNA-RACK1 in six-well plates at a density of 1000 cells/well. After approximately two weeks, cells were fixed with 4% paraformaldehyde for 20 min and stained with 0.1% crystal violet. Only colonies with more than 50 cells were counted under a microscope. The same procedure was performed in triplicate.

### 2.5. Wound Healing Assay

U87 and U251 cells were plated in six-well plates and transfected with 100 nM NC or siRNA-RACK1 when reaching 80%–90% density. A wound was made using a 1 mL plastic pipette tip, then cells were washed with phosphate-buffered saline (PBS) two times and cultured in a medium containing 2% fetal bovine serum (FBS) at 37 °C. The size of wound was measured under a microscope at 0 and 24 h after wounding.

### 2.6. Transwell Invasion Assay

To measure cell invasion, 24-well transwell chambers (Corning Inc., Tewksbury, MA, USA) were used. Matrigel (BD Bioscience, Bedford, MA, USA) was diluted at 1:8 with cold DMEM without FBS and coated in the upper compartment chamber. Then, different groups of cells (2 × 104) were plated into the upper wells with 100 µL serum‑free DMEM, and the bottom chamber filled with DMEM containing 10% FBS. After the cells were incubated at 37 °C for 24 h, non-invasive cells on the top chambers were gently wiped with cotton wool. Invasive cells on the bottom surface were fixed with 4% paraformaldehyde for 0.5 h and stained with 0.2% crystal violet for two hours, before they were counted under a light microscope.

### 2.7. Real-Time PCR Analysis

The mRNA expression of RACK1 in glioma tissues and cell lines was detected by RT-PCR. Total RNA was extracted using the trizol reagent (Invitrogen, Carlsbad, CA, USA), and first-strand cDNA was synthesized from 1 μg of total RNA using a Primescript™ RT reagent kit (TakaRa, Dalian, China) according to the manufacturer's instructions. RT-PCR was carried out using a standard SYBR Green II PCR kit (TakaRa, Dalian, China) on a LightCycler480 (Roche, San Francisco, CA, USA). Specific primers for RACK1 were: sense: 5’-CCGGCAGATTGTCTCTGGAT-3’; antisense: 5’-CGGACACAAGACACCCACTC-3’, and specific primers for β-actin were: sense: 5’-TATGCCAACACAGTGCTGTC-3’; antisense: 5’-GCTCAGGAGGAGCAATGATC-3’. The relative changes of RACK1 mRNA expression was normalized with β-actin expression level and calculated using the 2^−△△Ct^ method. The experiments were performed in triplicate.

### 2.8. Western Blot Analysis

U87 and U251 cells (control, NC, siRNA1, and siRNA2-RACK1) were transfected for 72 h and collected. Total proteins were extracted using cold Radio Immunoprecipitation Assay (RIPA) buffer with protease inhibitor cocktail tablet (Roche, San Francisco, CA, USA) and phosphatase Inhibitor Cocktail 2 (Sigma, New York, NY, USA) for 30 min on ice, and centrifuged at 12,000× *g* for 20 min at 4 °C. According to the manufacturer’s recommendations, protein concentrations were examined by Bicinchoninic Acid (BCA) protein assay kit (Thermo, Waltham, MA, USA). Proteins (40 μg/lane) were separated by 10% sodium dodecyl sulfate-polyacrylamide gel electrophoresis (SDS-PAGE) and then transferred onto polyvinylidene fluoride (PVDF) membranes (Millipore, Billerica, MA, USA). The primary antibody to RACK1, MMP2, MMP9, ZEB1, N-Cadherin, Integrin β1, Cyclin D1, CDK6, and β-actin was incubated with the membranes overnight at 4 °C. After incubation with secondary antibodies (Jackson Immuno Research, West Grove, PA, USA; 1:5000) at room temperature for 2.5 h, the specific protein was visualized using Enhanced chemiluminescence (ECL, GE Healthcare, Mliwaukee, WI, USA) and quantified by Quantity One software (Bio‑Rad, Hercules, CA, USA). All of the Western blots were performed at least three times. Antibodies against RACK1, MMP2, MMP9, ZEB1, N-Cadherin, Cyclin D1, CDK6, as well as Integrin β1 were obtained from CST Biotech (Cell Signaling Technology, Boston, MA, USA; 1:1000). Antibody to β-actin was purchased from Sigma (New York, NY, USA).

### 2.9. Immunohistochemistry (IHC)

The expression of RACK1 was detected by IHC in 65 cases of glioma tissues. Paraffin tissues of glioma were cut into 5 μm sections and then evaluated for the expression of RACK1. The whole process was performed according to the standard protocols. Primary antibody was diluted at 1:100 (Santa Cruz Biotechnology, Santa Cruz, CA, USA). The IHC results were evaluated by two pathologists independently, according to the percentage of RACK1 positive cells in microscopic fields at ×400.

### 2.10. Flow Cytometry Analysis

For cell cycle analysis, transiently transfected U87 and U251 cells were cultured in six-well plates. All cells were harvested by trypsinization after 48 h, washed with ice-cold PBS two times, and then fixed with 75% ethanol at 4 °C overnight. The cells were resuspended in 200 μL PBS with 5 μL RNase and 10 μL propidium iodide (PI) (Beyotime Institute of Biotechnology, Shanghai, China) for 25 min in the dark at room temperature. Cell cycle distribution was evaluated by FC500 flow cytometry (Beckman Coulter, Brea, CA, USA).

A cell apoptosis assay was carried out by using Annexin V-FITC/propidium iodide (PI) Apoptosis Detection Kit (BD Biosciences, San Jose, CA, USA) according to the manufacturer’s recommendations. Briefly, U87 and U251 cells transfected with siRNA1-RACK1, siRNA2-RACK1, or si-NC were harvested after 72 h for apoptosis analysis. A total of 195 μL of binding buffer, 5 μL of Annexin V-FITC, and 3 μL of propidium iodide (PI) were added to the suspension, and cells were incubated for 10 min at room temperature in the dark. The cells were immediately examined by flow cytometry (Beckman Coulter, Atlanta, GA, USA). All experiments were conducted independently at least three times.

### 2.11. Statistical Analysis

Statistical analyses were conducted using SPSS software, version 20.0 (IBM, SPSS, Chicago, IL, USA). Data was presented as mean ± SD. Differential expression of RACK1 between glioma and normal brain tissues was detected by independent two sample *t*-test. Overall survival curves were plotted from Kaplan-Meier estimates, while log-rank tests were conducted to compare the survival distributions between two groups. Differences were considered statistically significant if the *p* value was less than 0.05.

## 3. Results

### 3.1. Overexpression of RACK1 Correlates with Histological Malignancy and Poor Prognosis in Human Gliomas

**Figure 1:**
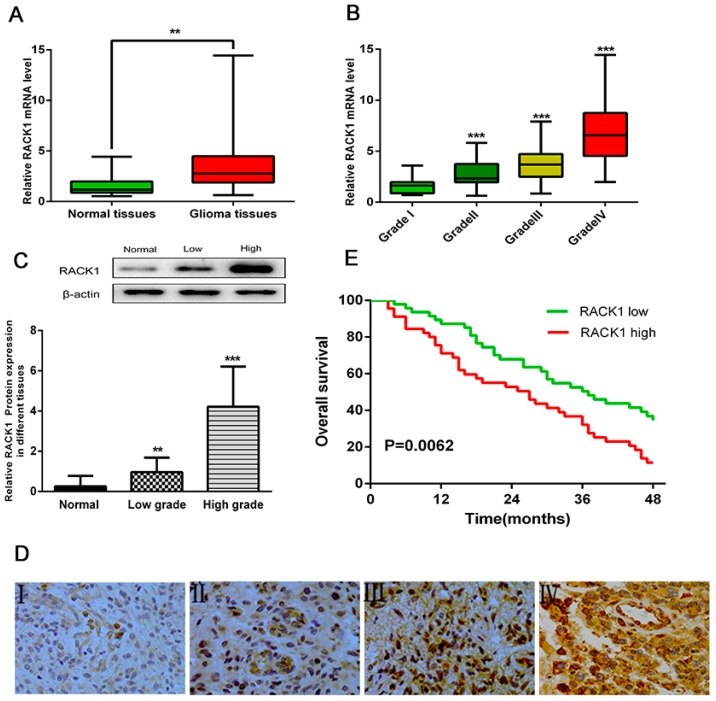
Overexpression of RACK1 was correlated with histological malignancy and poor clinical outcome in human gliomas. (**A**) The mRNA expression levels of RACK1 in glioma and normal brain tissues were detected by RT-PCR assays (** *p* < 0.01); (**B**) RACK1 mRNA expression levels in different glioma tissues (*** *p* < 0.001, *** *p* < 0.001, *** *p* < 0.001 as compared with grade I glioma tissues); (**C**) The protein levels of RACK1 in normal and glioma brain tissues (** *p* < 0.01, *** *p* < 0.001 as compared with normal brain tissues); (**D**) The expression of RACK1 was examined by immunohistochemistry staining in different grades of glioma tissues; (**E**) Kaplan-Meier analysis was performed to investigate the association of RACK1 expression level with the overall survival (OS) time in 92 cases of glioma patients (*p* = 0.0062). (** *p* < 0.01, *** *p* < 0.001).

To explore whether RACK1 could be an important factor in the development and progression of glioma, we first evaluated its mRNA expression level in glioma tissues (*n* = 173) and normal brain tissues (*n* = 20) by RT-PCR, discovering that RACK1 expression was statistically up-regulated in glioma tissues compared with normal brain tissues (Figure 1A, ** *p* < 0.01). Furthermore, we investigated the relationship between RACK1 expression level and histological malignancy in 32 cases of grade I, 58 cases of grade II, 49 cases of grade III, and 34 cases of grade IV glioma tissues. As illustrated in Figure 1B, with the increase of RACK1 expression, the malignancy of glioma showed an increasing tendency (*** *p* < 0.001, *** *p* < 0.001, *** *p* < 0.001 as compared with grade I glioma tissues).

The up-regulation of RACK1 in glioma tissues was further confirmed by Western blot and immunohistochemical staining (IHC). For Western blot, we used 10 cases of normal brain tissues, 50 cases of low grade (I–II grade), and 30 cases of high grade (III–IV grade) malignancy glioma tissues, finding that the protein level of RACK1 was dramatically higher in high degree and low degree malignancy glioma tissues than in normal brain tissues (Figure 1C, ** *p* < 0.01, *** *p* < 0.001 as compared with normal brain tissues; Figure S1).

The expression of RACK1 was further detected by IHC in another 65 cases of glioma tissues. We observed positive immunoreactivity of RACK1 in different grades of human glioma tissues. The expression of RACK1 was 3.33% (7/211) for grade I, 12.87% (22/171) for grade II, 30.19% (48/159) for grade III, and 51.89% (96/185) for grade IV glioma tissues, as shown in Figure 1D, indicating that the RACK1 protein was greatly up-regulated in high grade compared with the corresponding low grade glioma tissues.

### 3.2. Upregulation of RACK1 Is Associated with Poor Prognosis of Glioma Patients

In order to further determine the prognostic significance of RACK1 in glioma, Kaplan–Meier survival analysis was performed to evaluate the relationship between RACK1 expression levels and overall survival rates in 92 glioma cases, discovering that patients with high RACK1 expression had a significantly worse prognosis than those with low expression levels (Figure 1E, *p* = 0.0062, HR = 1.898, 95% CI: 1.225–3.203). The data suggested that RACK1 was generally and dramatically up-regulated in glioma tissues, probably correlated with poor prognosis in glioma cases.

### 3.3. Silencing of RACK1 Expression in Glioma Cells by siRNA

**Figure 2:**
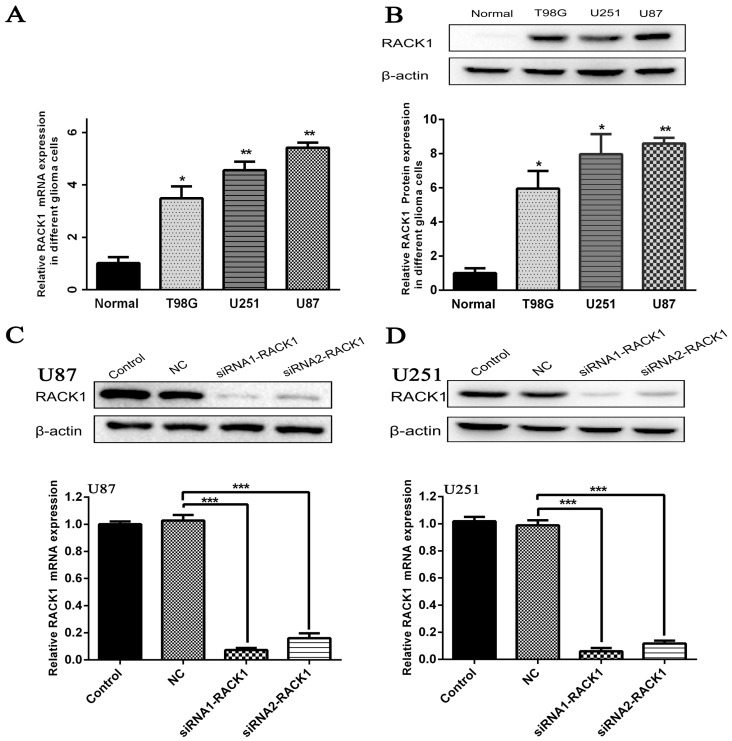
Silence of RACK1 expression in U87 and U251 cells by RACK1-siRNAs. (**A**,**B**) The mRNA and protein expression levels of RACK1 in normal tissues and three glioblastoma cell lines (T98G, U251, and U87) were detected by RT-PCR and Western blot; (**C**,**D**) The silencing effect of the siRNA1-RACK1 and siRNA2-RACK1 was confirmed by Western blot and RT-PCR in U87 and U251 cells. (* *p* < 0.05, ** *p* < 0.01, *** *p* < 0.001).

To investigate the functional role of RACK1 in glioma, we measured its mRNA and protein expression levels in normal tissues and three glioblastoma cell lines (T98G, U87, and U251) with RT-PCR and Western blot. Our data suggested that the mRNA and protein expression levels of RACK1 were significantly higher in three glioblastoma cell lines than in normal brain tissues (Figure 2A * *p* < 0.05, ** *p* < 0.01, ** *p* < 0.01; Figure 2B * *p* < 0.05, * *p* < 0.05, ** *p* < 0.01).

Then siRNA1-RACK1 and siRNA2-RACK1 were transfected into U87 and U251 cells, which had the highest levels of RACK1. After transfection for 48 h, the expression of RACK1 was confirmed by RT-PCR and Western blot. As shown in Figure 2C,D RACK1-siRNAs could decrease RACK1 expression effectively in glioblastoma cells in U87 and U251 cells, respectively, as compared with NC groups (Figure 2C *** *p* < 0.01, *** *p* < 0.01 for U87; Figure 2D *** *p* < 0.01,*** *p* < 0.01 for U251).

### 3.4. Effects of RACK1 on U87 and U251 Cell Proliferation

**Figure 3:**
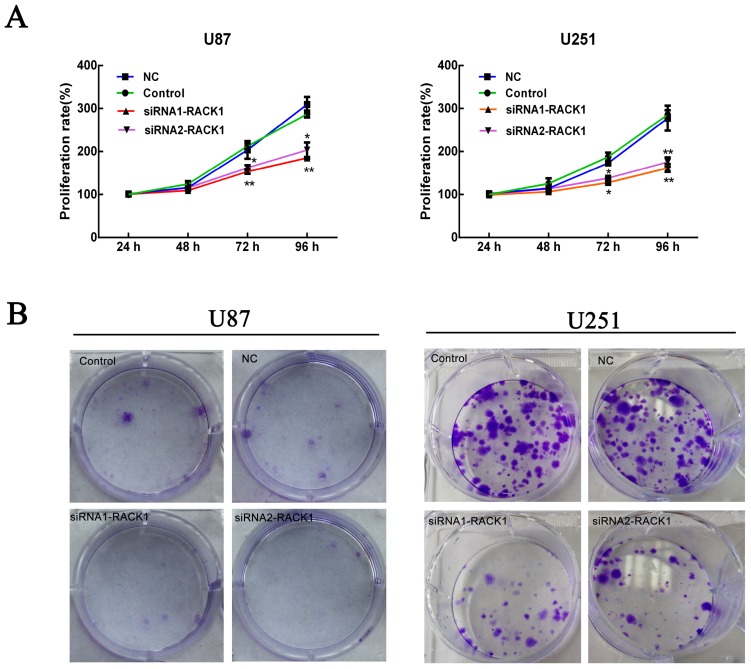
Knockdown of RACK1 suppressed the proliferation of U87 and U251 cells. (**A**) CellTiter 96 aqueous one solution reagent (MTS) assay demonstrated that the proliferation activities of the U87 and U251 cells were significantly inhibited by siRNA1-RACK1 and siRNA2-RACK1 at 96 h after transfection (** *p* < 0.01, * *p* < 0.05 for U87, ** *p* < 0.01, ** *p* < 0.01 for U251); (**B**) Colony formation assay indicated that silencing of RACK1 significantly decreased the number of colonies of the U87 and U251 cells in comparison with NC groups.

Cell proliferation was calculated via MTS and colony formation assays were performed after knockdown of RACK1 in U87 and U251 cells by siRNAs. As shown in Figure 3A, MTS assays demonstrated that siRNA1-RACK1 and siRNA2-RACK1 could significantly suppress the proliferation activity of the U87 and U251 cells in contrast with NC groups at 96 h after transfection (** *p* < 0.05 for U87; ** *p* < 0.01 for U251). In addition, a fifteen-day colony formation assay revealed that silencing of RACK1 drastically decreased the number of colonies formed in U87 and U251 cells compared with NC groups (Figure 3B).

**Figure 4:**
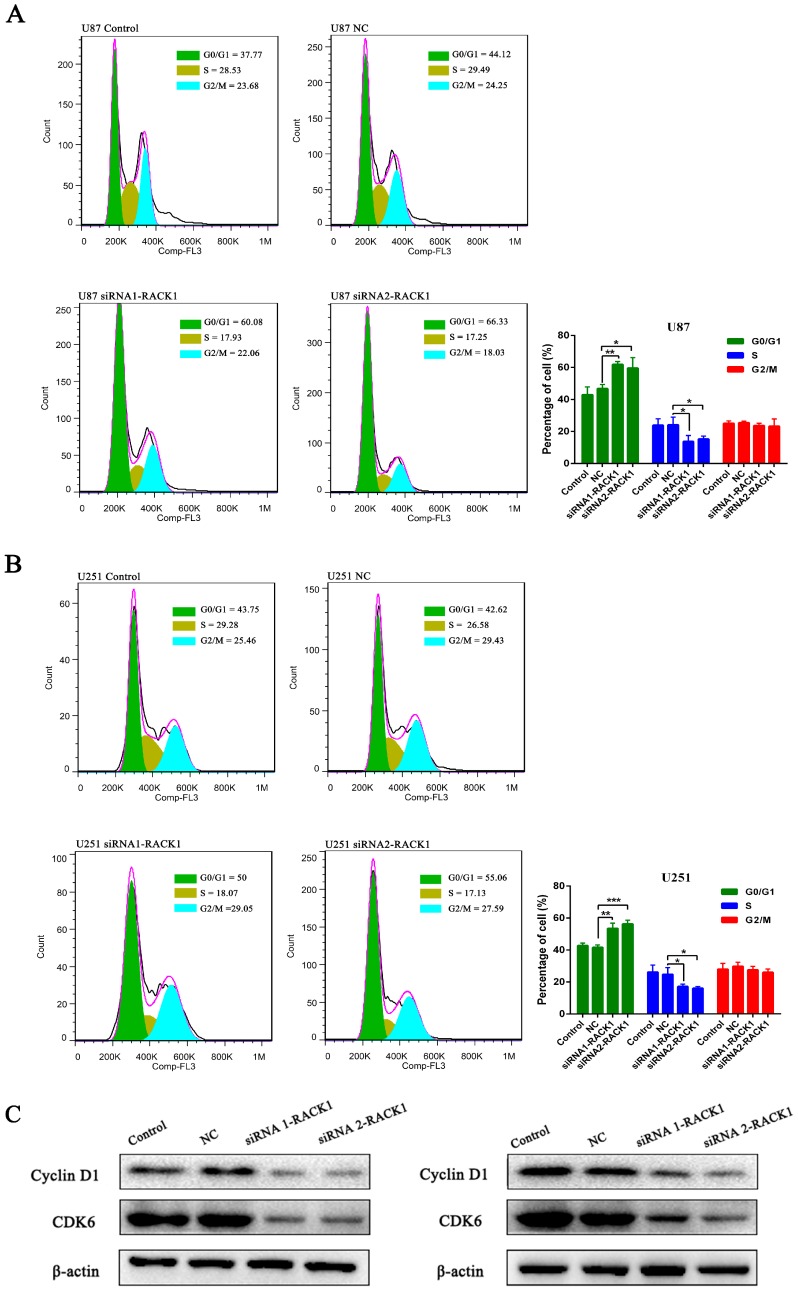
The effect of RACK1 on glioma cell cycle. (**A**,**B**) Down-regulation of RACK1 led to a dramatic accumulation of cells in G0/G1 phase and their obviously reduced proportions at the S phase (* *p* < 0.05, ** *p* < 0.01, *** *p* < 0.001); (**C**) Western blot analysis was performed to determine the expressions of Cyclin D1 and CDK6 both in U87 and U251 cell lines.

Subsequently, to investigate whether the repressive effect of RACK1-siRNAs in the proliferation of glioma cells was mediated by inhibiting cell cycle progression or promoting apoptosis, a flow cytometry analysis was performed. The results demonstrated that knockdown of RACK1 led to a dramatic accumulation of cells in G0/G1 phase (* *p* < 0.05 for U87; * *p* < 0.05 for U251), while obviously reduced their proportions at the S phase in comparison with NC groups both in U87 and U251 cells (Figure 4A,B), implying the potential acceleration of the cell cycle for glioma by high RACK1 levels.

To further explore the possible mechanism by which RACK1 promoted the glioma cell cycle, we then investigated the protein levels of G1/S transition key regulators, such as Cyclin D1 and CDK6. As shown in Figure 4C, after knockdown of RACK1 for 72 h, the expression of Cyclin D1 and CDK6 was greatly reduced compared with the NC groups both in U87 and U251 cells, which indicated that RACK1 might exert its functions through an activated G1/S cell cycle transition signaling pathway. These results supported the idea that overexpression of RACK1 played a crucial role in glioma growth and progression.

**Figure 5:**
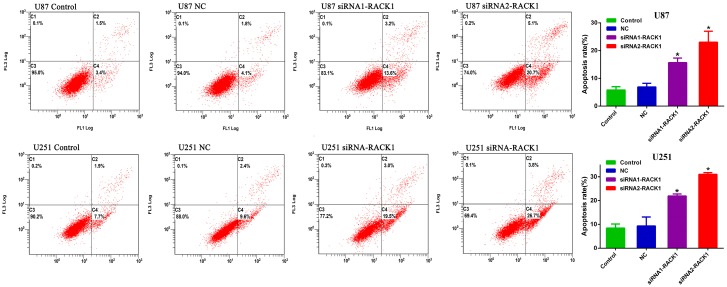
Annexin V-FITC/PI staining and flow cytometry analysis demonstrated that knockdown of RACK1 dramatically enhanced the apoptosis rate compared with negative control (NC) groups both in U87 and U251 cells. * *p* < 0.05.

As apoptosis regulation was a potential contributing factor to tumor development and progression, we then examined the effects of RACK1 on the apoptosis of glioma cells by Annexin V FITC and PI double labeled (Annexin V-FITC/PI) staining assay. As shown in Figure 5, the proportions of apoptotic cells transfected with RACK1-siRNAs were significantly increased compared with those in the NC groups (* *p* < 0.05, * *p* < 0.05 for U87, * *p* < 0.05, * *p* < 0.05 for U251). These findings implied that down-regulation of RACK1 could inhibit tumor cell proliferation via promoting apoptosis in glioma.

### 3.5. Downregulation of RACK1 Inhibited the Migration and Invasion of Glioma Cells

**Figure 6:**
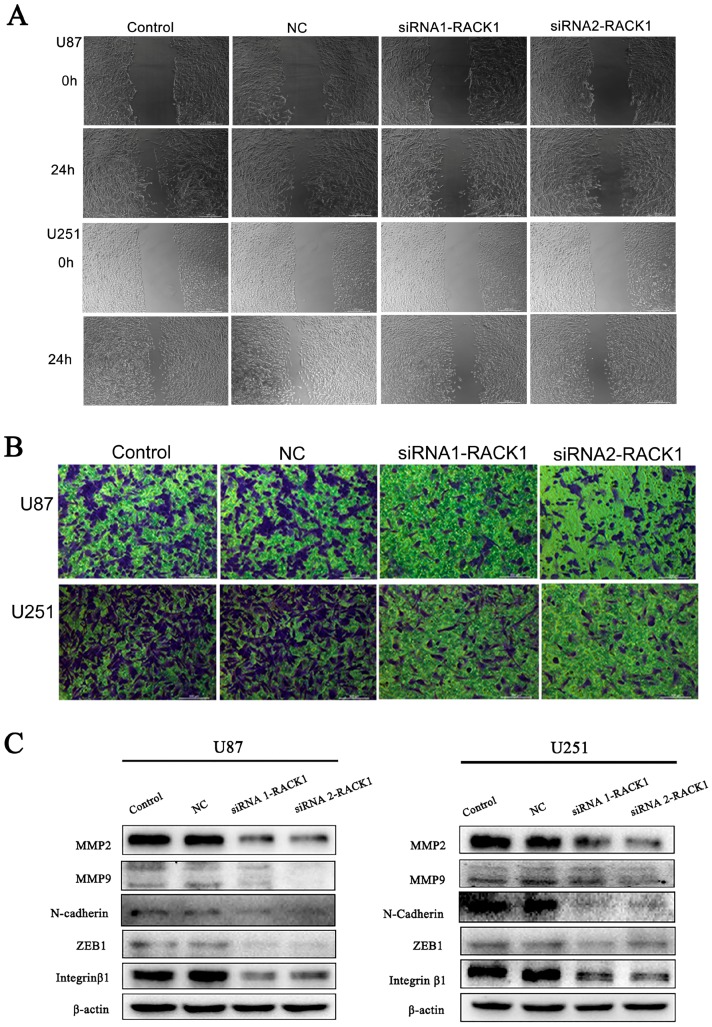
Downregulation of RACK1 inhibited the migration and invasion of glioma cells by suppressing epithelial-mesenchymal transition (EMT) markers. (**A**,**B**) Wound healing and transwell assays demonstrated that siRNA1-RACK1 and siRNA2-RACK1 could significantly inhibit cell migration and invasion in U87 and U251 cell lines, respectively, compared with NC groups; (**C**) Western blot analysis was performed to determine the expressions of EMT markers, such as MMP2, MMP9, N-Cadhenin, ZEB1, and Integrin β1 in the U87 and U251 cell lines. Scale bar: 100 µm.

Wound healing and transwell assays were performed to investigate the functional role of RACK1 in migration and invasion of glioma cells. In the wound healing assay, compared with NC groups, siRNA1-RACK1 and siRNA2-RACK1 significantly inhibited cell migration after 24 h of incubation (Figure 6A). Our results of the following transwell migration assays showed that most of the U87 and U251 cells invaded from the top to the bottom chambers in control and NC groups, but not in the siRNA1-RACK1 and siRNA2-RACK1 groups, with the quantification of cells on the bottom chamber markedly decreased in the RACK1-siRNAs groups (Figure 6B). Collectively, these findings indicated that RACK1 played a significant functional role in the regulation of glioma cell migration and invasion.

During tumorigenesis, the epithelial-mesenchymal transition (EMT) plays a crucial role in migration and invasion of various cancers, and the change of EMT protein levels was considered an important mechanism for the increased motility of glioma cancer cells. Therefore, we examined the expression of well-recognized EMT markers, such as MMP2, MMP9, ZEB1, N-cadherin, and Integrin β1. As shown in Figure 6C, we found that the expression levels of MMP‑2, MMP‑9, ZEB1, N-cadherin, and Integrin β1 significantly diminished in the siRNA1-RACK1 and siRNA2-RACK1 groups, suggesting that siRNA-RACK1 might suppress migration and invasion of malignant glioma cells by inhibiting the EMT signaling pathway.

## 4. Discussion

Recently, an increasing number of researches demonstrated that aberrant expression of RACK1 was supposed to be prevalent in a wide spectrum of cancers, such as breast cancer [15,26], hepatocellular carcinoma [27], and gastric cancer [28], suggesting that abnormal expression of RACK1 might be correlated with cancer development and progression.

Malignant gliomas are the most primary and lethal brain tumors. The abnormal expression of certain molecules has potential significance for clinical application in diagnostic and prognostic evaluation of gliomas. The promoter methylation of *O*-6-methylguanine-DNA methyltransferase (MGMT) and IDH-1 have been designated as meaningful molecular biomarkers for glioma in the National Comprehensive Cancer Network (NCCN) guidelines [29]. Peng et al. also reported that abnormal expression of RACK1 were associated with glioma progression by RT-PCR in 45 glioma tissues [30]. However, very few reports exist so far showing abnormal RACK1 expression at the protein level in glioma, with immunohistochemical data only available for breast cancer [15], gastric cancer [18], and esophageal squamous cell carcinoma [24].

In this study, we increased the sample size to 193 cases for RT-PCR, 90 cases for Western blot, and another 65 cases of glioma tissues were evaluated immunohistochemically to continually study the relationship between the overexpression of RACK1 and gliomas in depth. We demonstrated that the mRNA expression level of RACK1 in glioma tissues was significantly higher than that in normal brain tissues, which was consistent with the research results by Lin et al. and Guo et al. in epithelial ovarian cancer and hepatocellular carcinoma [20,27]. Moreover, with the increase of RACK1 mRNA expression, the malignancy of glioma showed an increasing tendency, and the expression level of RACK1 in grade IV was 4.27 times higher than grade I glioma tissues. The most surprising finding was that we observed fold changes of RACK1 protein levels in low grade (I–II grade) and high grade (III–IV grade) malignancy groups (3.69 and 16.22 compared with normal brain tissues, respectively) in Western blot analysis. Therefore, we conclude that high levels of RACK1 were correlated with the progression of glioma.

Overexpression of RACK1 has been strongly linked to advanced clinical stage and poor prognosis in a variety of solid cancers, and it was especially accurate as a prognostic indicator in breast cancer [26], but its prognostic value in glioma remained unclear. In this study, we performed Kaplan-Meier survival analyses and log-rank tests to evaluate the association between RACK1 expression levels and survival outcomes of glioma patients for the first time, discovering that patients with high RACK1 expression had notably shorter overall survival time than those with low RACK1 expression, which was compatible with the research results by Cao et al. and Li et al. that up-regulated expression of RACK1 promoted tumor growth and predicted poor prognosis in breast cancer and pancreatic ductal adenocarcinoma patients [26,31]. These data suggested that overexpression of RACK1 might be a candidate clinical diagnostic biomarker and negative prognostic indicator for patients with glioma.

In addition, function and mechanism research was conducted in cell models to find out the crucial role of RACK1 in glioma. Ruan et al. and Shen et al. provided evidence that highly expressed RACK1could enhance the proliferation of hepatocellular carcinoma and prostate cancer [23,32]. Similar results were presented in our study by MTS assay that RACK1-siRNAs drastically suppressed the proliferation activity both in U87 and U251 cells in contrast with NC cells. Moreover, colony formation assays also revealed that silencing of RACK1 greatly decreased the number of colonies formed. Additionally, knockdown of RACK1 led to a significant accumulation of cells in G0/G1 phase and correspondingly decreased the percentage of cells in S phase, and also promoted cell apoptosis. We also firstly discovered that the protein levels of G1/S transition key regulators, Cyclin D1 and CDK6, were remarkably reduced when RACK1 was silenced both in U87 and U251 cells. Concordantly with our results, Li et al. reported that RACK1 was highly expressed in pancreatic ductal adenocarcinoma (PDAC) and could induce G1/S cell cycle arrest by decreasing the expression of Cyclin D1 [31], while Zhang et al. reported that overexpression of RACK1 contributed to the progression of oral squamous cell carcinoma (OSCC) and stable silencing of RACK1 resulted in a distinct G1 and G2 phase arrest by downregulating Cyclin B1 and Cyclin D1 [33].

Some researchers have found that RACK1 not only participated in the morphology characteristic of adhesion through the formation of focal adhesions and stress fibers in some cancer cells, but also played an important role in cell migration [34,35]. More recently, the EMT process had been described as crucial for cancer metastasis progression in which RACK1 could participate [36]. Wang et al. discovered that silencing of RACK1 greatly suppressed the protein level of Vimentin, as well as increased the expression of E-cadherin in esophageal squamous cell carcinoma [24]. In our study, we found that blocking the expression of RACK1 greatly inhibited migration and invasion ability of glioma cells, and discovered that knockdown of RACK1 significantly decreased the expression of EMT markers, such as MMP2 and MMP9, ZEB1, N-cadherin, and Integrin-β1. These data suggested that RACK1 played a pivotal role in glioma metastasis. However, the manner in which RACK1 regulated the genes related to cell cycle, apoptosis, migration, and invasion has not been fully clarified in this study, and needs to be elucidated by further well-designed studies in the future.

## 5. Conclusions

In conclusion, our study demonstrated that RACK1 was dramatically elevated in glioma tissues and correlated with the histological malignancy of glioma. Furthermore, overexpression of RACK1 was tightly associated with poor prognosis outcome of glioma patients and promoted cell proliferation, migration, and invasion, as well as inhibited apoptosis of glioma cells. These data suggest that RACK1 might serve as an underlying prognostic factor and a new therapeutic target for the treatment of human glioma.

## Supplementary Materials

The following are available online at www.mdpi.com/1660-4601/13/10/1021/s1, Figure S1: Overexpression of RACK1 in glioma tissues was examined by Western-blot in 10 cases of normal brain tissues, 50 cases of low grade (I–II grade) and 30 cases of high grade (III–IV grade) malignancy of glioma tissues.

## References

[1] Armstrong T.S., Wen P.Y., Gilbert M.R., Schiff D. (2012). Management of treatment-associated toxicites of anti-angiogenic therapy in patients with brain tumors. Neuro-Oncology.

[2] Cai J.J., Qi Z.X., Hua W., Zhu J.J., Zhang X., Yao Y., Mao Y. (2014). Increased expression of Capn4 is associated with the malignancy of human glioma. CNS Neurosci. Ther..

[3] Yang T., Kong B., Kuang Y.Q., Cheng L., Gu J.W., Zhang J.H., Shu H.F., Yu S.X., He W.Q., Xing X.M. (2014). Overexpression of MACC1 protein and its clinical implications in patients with glioma. Tumour Biol..

[4] Stupp R., Mason W.P., van den Bent M.J., Weller M., Fisher B., Taphoorn M.J., Belanger K., Brandes A.A., Marosi C., Bogdahn U. (2005). Radiotherapy plus concomitant and adjuvant temozolomide for glioblastoma. N. Engl. J. Med..

[5] Zhu V.F., Yang J., Lebrun D.G., Li M. (2012). Understanding the role of cytokines in glioblastoma multiforme pathogenesis. Cancer Lett..

[6] Sathornsumetee S., Reardon D.A., Desjardins A., Quinn J.A., Vredenburgh J.J., Rich J.N. (2007). Molecularly targeted therapy for malignant glioma. Cancer.

[7] Tabatabai G., Hegi M., Stupp R., Weller M. (2012). Clinical implications of molecular neuropathology and biomarkers for malignant glioma. Curr. Neurol. Neurosci. Rep..

[8] Mochly-Rosen D., Khaner H., Lopez J. (1991). Identification of intracellular receptor proteins for activated protein kinase C. Proc. Natl. Acad. Sci. USA.

[9] McCahill A., Warwicker J., Bolger G.B., Houslay M.D., Yarwood S.J. (2002). The RACK1 scaffold protein: A dynamic cog in cell response mechanisms. Mol. Pharmacol..

[10] Adams D.R., Ron D., Kiely P.A. (2011). RACK1, a multifaceted scaffolding protein: Structure and function. Cell Commun. Signal..

[11] Daniels C.C., Rovnak J., Quackenbush S.L. (2008). Walleye dermal sarcoma virus Orf B functions through receptor for activated C kinase (RACK1) and protein kinase C. Virology.

[12] Hermanto U., Zong C.S., Li W., Wang L.H. (2002). RACK1, an insulin-like growth factor I (IGF-I) receptor-interacting protein, modulates IGF-I-dependent integrin signaling and promotes cell spreading and contact with extracellular matrix. Mol. Cell. Biol..

[13] Li J.J., Xie D. (2015). RACK1, a versatile hub in cancer. Oncogene.

[14] Wehner P., Shnitsar I., Urlaub H., Borchers A. (2011). RACK1 is a novel interaction partner of PTK7 that is required for neural tube closure. Development.

[15] Cao X.X., Xu J.D., Xu J.W., Liu X.L., Cheng Y.Y., Wang W.J., Li Q.Q., Chen Q., Xu Z.D., Liu X.P. (2010). RACK1 promotes breast carcinoma proliferation and invasion/metastasis in vitro and in vivo. Breast Cancer Res. Treat..

[16] Wang W.D., Wen Z., Ji W., Ma Y. (2015). RACK1 expression contributes to JNK activity, but JNK activity does not enhance RACK1 expression in hepatocellular carcinoma SMMC-7721 cells. Oncol. Lett..

[17] Campagne C., Jule S., Alleaume C., Bernex F., Ezagal J., Chateau-Joubert S., Estrada M., Aubin-Houzelstein G., Panthier J.J., Egidy G. (2013). Canine melanoma diagnosis: RACK1 as a potential biological marker. Vet. Pathol..

[18] Chen L., Min L., Wang X., Zhao J., Chen H., Qin J., Chen W., Shen Z., Tang Z., Gan Q. (2015). Loss of RACK1 promotes metastasis of gastric cancer by inducing a miR-302c/IL8 signaling loop. Cancer Res..

[19] Li J., Guo Y., Feng X., Wang Z., Wang Y., Deng P., Zhang D., Wang R., Xie L., Xu X. (2012). Receptor for activated C kinase 1 (RACK1): A regulator for migration and invasion in oral squamous cell carcinoma cells. J. Cancer Res. Clin. Oncol..

[20] Lin Y., Cui M., Teng H., Wang F., Yu W., Xu T. (2014). Silencing the receptor of activated C-kinase 1 (RACK1) suppresses tumorigenicity in epithelial ovarian cancer in vitro and in vivo. Int. J. Oncol..

[21] Atala A. (2015). Re: Ion channel TRPM8 promotes hypoxic growth of prostate cancer cells via an O2-independent and RACK1-mediated mechanism of HIF-1α stabilization. J. Urol..

[22] Berns H., Humar R., Hengerer B., Kiefer F.N., Battegay E.J. (2000). RACK1 is up-regulated in angiogenesis and human carcinomas. FASEB J..

[23] Ruan Y., Sun L., Hao Y., Wang L., Xu J., Zhang W., Xie J., Guo L., Zhou L., Yun X. (2012). Ribosomal RACK1 promotes chemoresistance and growth in human hepatocellular carcinoma. J. Clin. Investig..

[24] Wang N., Liu F., Cao F., Jia Y., Wang J., Ma W., Tan B., Wang K., Song Q., Cheng Y. (2015). RACK1 predicts poor prognosis and regulates progression of esophageal squamous cell carcinoma through its epithelial-mesenchymal transition. Cancer Biol. Ther..

[25] Choi Y.Y., Lee S.Y., Lee W.K., Jeon H.S., Lee E.B., Lee H.C., Choi J.E., Kang H.G., Lee E.J., Bae E.Y. (2015). RACK1 is a candidate gene associated with the prognosis of patients with early stage non-small cell lung cancer. Oncotarget.

[26] Cao X.X., Xu J.D., Liu X.L., Xu J.W., Wang W.J., Li Q.Q., Chen Q., Xu Z.D., Liu X.P. (2010). RACK1: A superior independent predictor for poor clinical outcome in breast cancer. Int. J. Cancer.

[27] Guo Y., Wang W., Wang J., Feng J., Wang Q., Jin J., Lv M., Li X., Li Y., Ma Y. (2013). Receptor for activated C kinase 1 promotes hepatocellular carcinoma growth by enhancing mitogen-activated protein kinase kinase 7 activity. Hepatology.

[28] Deng Y.Z., Yao F., Li J.J., Mao Z.F., Hu P.T., Long L.Y., Li G., Ji X.D., Shi S., Guan D.X. (2012). RACK1 suppresses gastric tumorigenesis by stabilizing the β-catenin destruction complex. Gastroenterology.

[29] Van Meir E.G., Hadjipanayis C.G., Norden A.D., Shu H.K., Wen P.Y., Olson J.J. (2010). Exciting new advances in neuro-oncology: The avenue to a cure for malignant glioma. Cancer J. Clin..

[30] Peng R., Jiang B., Ma J., Ma Z., Wan X., Liu H., Chen Z., Cheng Q., Chen R. (2013). Forced downregulation of RACK1 inhibits glioma development by suppressing Src/Akt signaling activity. Onco. Rep..

[31] Li X., Xiao Y., Fan S., Xiao M., Wang X., Chen X., Li C., Zong G., Zhou G., Wan C. (2016). RACK1 overexpression associates with pancreatic ductal adenocarcinoma growth and poor prognosis. Exp. Mol. Pathol..

[32] Shen F., Yan C., Liu M., Feng Y., Chen Y. (2013). RACK1 promotes prostate cancer cell proliferation, invasion and metastasis. Mol. Med. Rep..

[33] Zhang X., Liu N., Ma D., Liu L., Jiang L., Zhou Y., Zeng X., Li J., Chen Q. (2016). Receptor for activated C kinase 1 (RACK1) promotes the progression of OSCC via the Akt/mTOR pathway. Int. J. Oncol..

[34] O’Donovan H.C., Kiely P.A., O’Connor R. (2007). Effects of RACK1 on cell migration and IGF-I signalling in cardiomyoctes are not dependent on an association with the IGF-IR. Cell Signal..

[35] Kiely P.A., Leahy M., O’Gorman D., O’Connor R. (2005). RACK1-mediated integration of adhesion and insulin-like growth factor I (IGF-I) signaling and cell migration are defective in cells expressing an IGF-I receptor mutated at tyrosines 1250 and 1251. J. Biol. Chem..

[36] Vincent-Salomon A., Thiery J.P. (2003). Host microenvironment in breast cancer development: Epithelial-mesenchymal transition in breast cancer development. Breast Cancer Res..

