# Supplementary material for: Overexpression of RACK1 Promotes Metastasis by Enhancing Epithelial-Mesenchymal Transition and Predicts Poor Prognosis in Human Glioma
Source: Int J Environ Res Public Health. 2016 Oct 18;13(10):1021. doi: 10.3390/ijerph13101021 (PMC5086760; doi:10.3390/ijerph13101021)
Supplement: Supplementary file 1 [file ijerph-13-01021-s001.pdf]

Int. J. Environ. Res. Public Health **2016**, *13*, 1021; doi:10.3390/ijerph13101021

Supplementary Materials: Overexpression of RACK1 Promotes Metastasis by Enhancing Epithelial-Mesenchymal Transition and Predicts Poor Prognosis in Human Glioma

Qiao-Li Lv, Yuan-Tao Huang, Gui-Hua Wang, Yan-Ling Liu, Jin Huang, Qiang Qu, Bao Sun, Lei Hu, Lin Cheng, Shu-Hui Chen and Hong-Hao Zhou

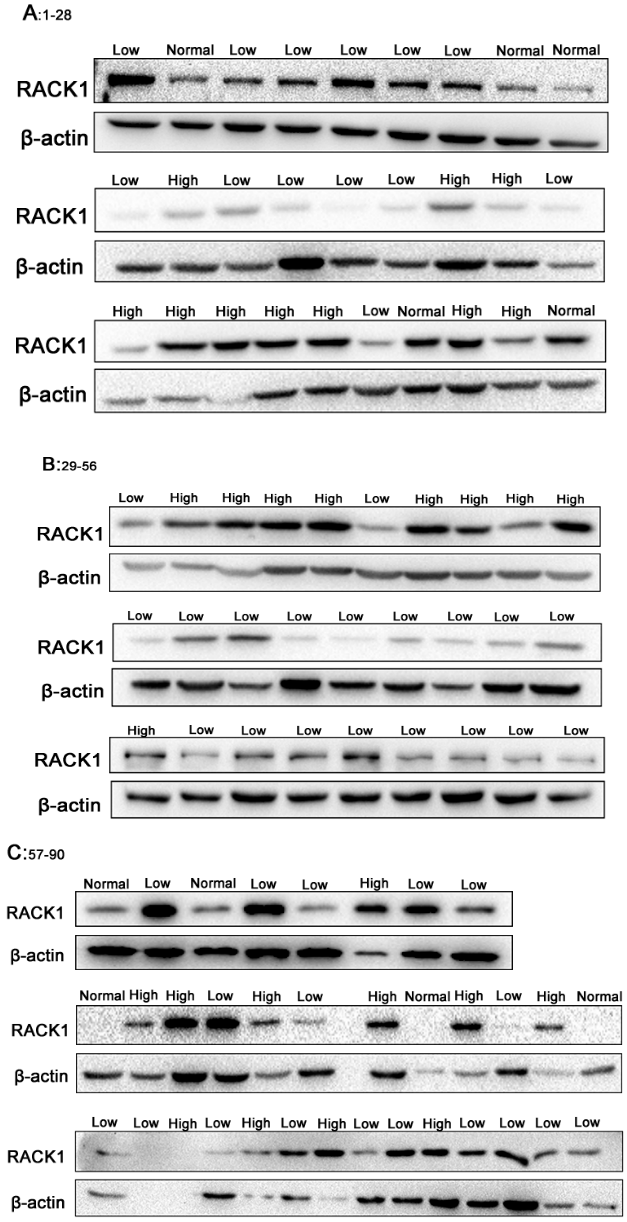

Figure 1. Protein levels of RACK1 in glioma tissues. (**A**,**B**,**C**) Overexpression of RACK1 in glioma tissues was examined by Western-blot in 10 cases of normal brain tissues, 50 cases of low grade (I–II grade) and 30 cases of high grade (III–IV grade) malignancy of glioma tissues.

© 2016 by the authors; licensee MDPI, Basel, Switzerland. This article is an open access article distributed under the terms and conditions of the Creative Commons by Attribution (CC-BY) license (http://creativecommons.org/licenses/by/4.0).
